# Telomere Maintenance and Oncogenesis

**DOI:** 10.3390/ijms262210941

**Published:** 2025-11-12

**Authors:** Antonio Torres-Montaner

**Affiliations:** Independent Researcher, 22400 Monzon, Spain; atorresmontaner@gmail.com

**Keywords:** telomeres, Rap1, Zscan4, PI3K/AKT pathway

## Abstract

An overwhelming majority of cancers exhibit telomere length reduction and differentiation markers consistent with a post-stem cell of origin. On the other hand, telomere shortening/damage is believed to protect cells from malignant transformation through induction of apoptosis. However, increased cancer incidence in the absence of apoptotic factors like p53 may suggest a favorable role of telomere shortening/damage in cancer development. Some findings suggest that telomere shortening may induce architectural changes in telomeric chromatin, such as those underlying the telomere position effect that support telomere maintenance of some tumors Here, we propose that several signaling pathways, in conjunction with telomere shortening/damage, may result in the release of Rap1 from telomeres. Its subsequent interaction with the embryonic stem cell marker Zscan4 may support immortalization and malignant transformation of the target cell.

## 1. Introduction

In the quest to understand the nature of cancer, a point of departure is provided by one of the most fundamental aspects of living organisms: the capacity for regeneration and self-renewal, which is also a hallmark of cancer. This property is compartmentalized within organisms and linked to germinal structures or cells endowed with embryonic features that maintain a capacity for regeneration. This idea led Conheim [1] to propose that cancer originates in embryonic remnants that persist into adult life, disconnected from their original sources, and later undergo aberrant growth. Later, histological research established that specialized somatic cells present in every tissue, initially known as reserve cells and later as normal stem cells, are responsible for replenishing the population of tissue cells, which are continually lost to wear and tear or shed after multiple rounds of proliferation and differentiation. The ability to indefinitely self-renew, which is shared by the so-called reserve cells and cancer cells, with its dual physiological and pathological functions, led to the assumption that cancer growth arises from a failure of tissue stem cells to respond to tissue regulatory signals that maintain normal tissue architecture. In other words, cancer originates directly from tissue stem cells. The modern concept of cancer stem cells assumes this hierarchical organization, ascribing embryonic/stem features to these cells and generally disregarding the concept of an origin in embryonic rests. However, this feature is often interpreted to preclude an origin of cancer in committed cells.

Regeneration potential is lost as soon as cells leave the stem cell compartment. This was first confirmed in the hematopoietic system. Even in the transition from long-term hematopoietic stem cells (LT-HSCs) to short-term hematopoietic stem cells (ST-HSCs), there is some loss of regeneration potential. In the next stage, transit-amplifying cells, which expand by continuous cell division, lose their capacity for indefinite self-renewal [2]. Similar changes occur in other tissues. Muscle satellite stem cells have been shown to lose their ability to self-renewal indefinitely immediately after their first division. “Pax7^+^ Myf5^−^ stem cells that undergo asymmetric self-renewal divisions on muscle fibers give rise to a basal Pax7^+^ Myf5^−^ stem cell daughter and an apical Pax7^+^ Myf5^−^ satellite cell with a more restricted proliferative potential” [3]. A similar early loss of regeneration potential is seen in other tissues, such as the mammary epithelium [4]. Thus, tissue homeostasis relies on the regeneration potential of tissue stem cells, which is supported by permanent telomerase activity. Upon exit from the stem cell compartment, regeneration potential and telomerase activity both decline in conjunction with telomere shortening and tissue differentiation/diversification. Reactivation of telomerase expression in committed cells should not disturb population dynamics, provided it is followed by a gradual decline in telomerase in coordination with cell differentiation/diversification. The loss of these features may lead to a perturbation of population dynamics, resulting in cancer. The presence of short/damaged telomeres preceding cancer suggests a facilitating role of telomere dysfunction in carcinogenesis and a predominant origin of cancer in committed cells. This concept is not at odds with the protective role of senescence induced by telomere shortening. In fact, telomere shortening is a double-edged sword. Sensing of telomere shortening by p53 leads to senescence, thereby protecting against cancer development. Through senescence, the inherent tendency of telomere shortening to reactivate telomerase or other means of telomere maintenance (ALT), a pre-requisite for cancer development, is abolished [5]. However, the DNA damage response in aging- and stress-induced senescence preferentially impacts the telomere region [6]. Cancer stem cells usually possess shorter telomeres than surrounding cells, consistent with a facilitating role of telomere shortening in cancer. Concomitantly, the telomere length of transformed cells is stabilized, which suggests that a sudden change in telomerase control occurs at the moment of transformation. Conversely, a direct origin of cancer in normal stem cells appears to be quite infrequent, which could be attributed to a role of telomerase in genomic and telomere protection. These concepts have been presented and discussed at length in previous publications [7,8,9,10]. In this study, we examine select oncogenic signaling pathways that may cooperate with telomere shortening/damage to promote cancer. In this process, inducing the malignant transformation entails the reactivation of telomerase expression and telomere maintenance under new types of control that include ALT or the reactivation in committed cells of latent genes that are physiologically active in embryonic development.

## 2. Normal Stem Cells and Oncogenic Pathways

It is difficult to maintain stem cells in an undifferentiated state in culture. It is only possible when they remain anchored to their niche. Oncogenes such as Ras can decouple cell cycle control from adhesion to the niche, leading to anchorage-independent growth, which is one of the earliest events in transformation [11]. At the same time, signaling pathways involved in oncogenesis trigger some degree of differentiation and exit from the stem cell compartment. In addition, most oncogenes disturb normal coordination between cell proliferation and cell differentiation, which may potentiate telomere shortening/damage. These conditions should make committed cells the preferential targets of malignant transformation. However, committed cells do not retain their capacity for indefinite self-renewal, which must be reacquired. This must coincide with a fundamental change in telomerase reactivation marked by telomere length stabilization.

The role of telomere erosion in cancer is reminiscent of a phenomenon observed in in vitro cell cultures known as crisis, where sustained telomere length reduction leads to massive apoptosis and then the emergence of cells with an immortal phenotype. For simplicity, we use the term telomere erosion/shortening to refer to the complex changes that telomeres and the telomere complex may undergo, as different routes of tumorigenesis may impact the telomere complex in addition to shortening.

Regarding the assumption that the process of transformation entails cell reprogramming and acquiring the ability to self-renew in a committed cell, it can be expected that the resulting cancer stem cell would exhibit a phenotype partially overlapping with that of a normal stem cell. In fact, although cancer stem cells are defined and isolated by surface markers shared with normal stem cells, they almost always express differentiation markers that allow for assignment to a particular stage in cell lineage. A widespread survey of CSCs identified in different cancer types confirmed this theory. Leukemia is a particularly relevant example as the origin of most leukemias can be traced to a block in differentiation, which imparts to the leukemia stem cell the phenotype corresponding to the specific stage of the developmental block [9].

The important role of telomere erosion in cancer predicts the preponderance of committed cell-derived cancers, but it does not exclude the existence of true stem cell cancers. The assumption of a stem cell origin, commonly attributed to cancers arising in highly proliferative tissues, is unlikely because the oncogenic pathways that drive these cancers consist of deregulated pathways that signal physiological differentiation on the same cells, placing them in the progenitor stage rather than the stem cell stage. Let us examine these pathways.

## 3. The Wnt Pathway and Crosstalk Between the Wnt/β-Catenin and AKT/mTOR Pathways

The Wnt pathway supports the self-renewal of stem and progenitor cells and is conspicuously expressed in highly regenerating tissues. catenin, the central player in this pathway, is maintained at a low level by GSK3β phosphorylation in a destruction complex composed of GSK3β, adenomatous polyposis coli (APC), Axin, CKI kinase, and β-catenin. Signaling by Wnt ligands acting on cell surface receptors of the Frizzled family inactivates GSK3β by phosphorylation at Serine 9 and blocks β-catenin destruction. This results in the accumulation of β-catenin, which is then translocated to the nucleus, where, in conjunction with TCF factors (mainly TCF4 in the intestine), it stimulates transcription of target genes expressed physiologically in the stem cell areas and proliferative compartment of different tissues and organs [12].

There is evidence of cross-talk between the Wnt/β-catenin pathway and the PI3-AKT-mTOR pathway, which might underlie some findings that we discuss later in this article. GSK3β is a critical node of both pathways. It has been shown that GSK3β inhibition and subsequent Wnt/β-catenin activation enhance HSC self-renewal. Concomitantly, GSK3β inhibition stimulates mTORC1, which promotes proliferation and differentiation. By simultaneously stimulating the Wnt pathway (enhancing self-renewal in HSCs) with inhibitors of GSK3β CHIR99021 or lithium and inhibiting the mTORC1 pathway with rapamycin, Huang et al. [13] were able to maintain long-term multilineage hematopoiesis in cytokine-free cultures treated with both inhibitors. Transplantation to secondary recipients confirmed the improved preservation of HSC function, corroborated by a higher degree of chimerism detected in cells treated with both inhibitors. This implies that Wnt signaling, activated in most instances through GSK3β inhibition, should drive some differentiation without the artificial inhibition of mTORC1. GSK3β is also inhibited by AKT [14] which, in turn, requires activity of the PI3K/AKT pathway, which is antagonized by PTEN. This is why the LT-HSC exhaustion caused by deregulated Wnt/β-catenin signaling is recapitulated by hyperactivation of the PI3K/AKT pathway, as observed after PTEN deletion. Here, we want to stress that mTORC1 activation through GSK·β inhibition associated with Wnt/β-catenin activation stimulates growth and, like c-Myc, promotes exit from the stem cell compartment. Stimulation of mTORC1 through deregulated Wnt signaling leads to engagement in cell proliferation and differentiation (exit from the stem cell compartment). This leads to stem cell exhaustion and decreased preservation of regenerating ability which likely correlates with telomere shortening.

Before addressing intestinal adenoma, it is important to remember that this type of lesion contains progenitor cells because Myc (an essential target of the Wnt/βcatenin pathway) accelerates exit from the stem cell compartment and detachment from the niche. This entails loss of stem cell regeneration potential, which is crucial for tumor maintenance and must be obtained from other sources. This has been demonstrated in hematopoietic tissue. According to Wilson et al., “the inability to down-regulate c-Myc resulted in increased differentiation at the expense of self-renewal. C-Myc-transduced cells repressed expression of N-cadherin and other cell adhesion molecules such as LFA-1 receptor and αL and β2-integrins accompanied by detachment to the niche and premature differentiation. Functionally, however, repopulation experiments showed that the repopulating ability of c-Myc transduced KLS-HSC cells was severely impaired. However, in addition to increased exit from the stem cell compartment, Myc overexpression may inhibit proper differentiation.” [15]. Similarly, p27 helps to maintain cells in an undifferentiated state, and it has been found that Skp2-mediated degradation of p27 is crucial for the progression of adenomas to carcinomas [16].

## 4. Adenoma–Carcinoma Sequence

Van de Wetering et al. extensively characterized the Wnt/β-catenin signature in the intestine. Downstream targets included CD44, BMP4, claudin-1, ENC1 and c-MYC, c-ETS2, and EPHB2. Cyclin D1, a previously known TCF-4 target, was not affected by dn-TCF-4. P21 was induced independently of p53 and marks the differentiated compartment, while p15, p19, and p16 were not expressed or were unaffected. Only c-MYC overrode the effect of dnTCF-4 on cell growth. It was established that c-MYC represses p21, and c-MYC and MIZ-1 bind to the p21 promoter [17].

Activating mutations of the Wnt pathway are the only known genetic alterations in early premalignant lesions in the intestine, such as aberrant crypt foci and small polyps [18,19]. In the hereditary syndrome known as hereditary polyposis, as well as in more than 85% of colorectal tumors, the initiating mutation is a loss-of-function mutation of APC, and the central oncogenic player is the nuclear accumulation of β-catenin and its binding to TCF molecules (mainly TCF4 in the intestine). Zhang et al. showed that hTERT is a target of the Wnt/β-catenin pathway. They also reported that silencing β-catenin suppressed telomerase activity, which was mediated by the suppression of TCF-4 [20].

The immediate consequence of deregulated β-catenin stimulation is the formation of a polyp or adenoma, which may transform into a fully malignant cancer over time. Although the rate of conversion from adenoma to cancer is not high, it is believed that most colorectal cancers arise from adenomas. Therefore, this type of lesion offers an opportunity to study the main steps in the conversion to malignancy.

Telomerase activity has been frequently evaluated in colorectal adenomas, and its incidence has varied from 0% to 100% in different studies. However, there is strong consensus that telomerase activity increases in parallel with the size of the polyp and its malignant potential. Tang [21] examined telomerase activity in 31 adenomatous polyps and 22 paired cancer–normal mucosa samples from patients with nonhereditary nonpolyposis colorectal cancer, finding a linear correlation between polyp progression and telomerase activity. The latter was detected in 18% of normal mucosa specimens, 16% of small (<1 cm) polyps, 20% of intermediate (>2 cm) polyps, 71% of large polyps, and 96% of adenocarcinomas, suggesting that telomerase reactivation correlates with progression of colorectal carcinogenesis.

A different study by Druliner [22] aimed to determine whether telomere length and telomere maintenance mechanisms could distinguish cancer-associated adenomas from those that are cancer-free. The author classified adenomas into two groups: adenomas adjacent to cancer (residual adenomas contiguous with cancer) (CAPs) and polyps not associated with cancer or cancer-free polyps (CFPs). Further, the author identified aggressive CAPs (if stage I–II cancer recurred or presented a stage IV polyp) and aggressive CFPs (if there was a recurrence of the polyp). The CAP polyps had shorter telomeres and higher telomerase reverse transcriptase (hTERT) expression compared to the CFPs. The degree of difference in hTERT expression between the polyps and normal colon tissue was significant for the CAP cases but not for the CFP cases, which could not be attributed to differences in the normal tissues. The aggressive CAPs exhibited the shortest range of telomere lengths, with the polyp tissue exhibiting the shortest telomeres. The study also measured the telomere lengths of peripheral blood leucocytes (PBLs) and normal colon mucosa. After adjustment for age, the CAP patients’ PBL telomeres were an average of 91.7 bp longer than those of the CFP patients, and the normal colon epithelium hosted significantly longer telomeres than PBL (we suggest that this indicates a relative loss of quiescence in normal stem cells, which decreases their regenerative potential and, paradoxically, their resistance to malignant transformation).

Plentz [23] used hematoxylin–eosin staining to measure telomere fluorescence intensity in consecutive histological sections from areas of high-grade dysplasia and adenoma. Signal intensity was weaker at the earliest morphologically defined stage of carcinoma–high-grade dysplasia with minimally invasive growth, suggesting that malignant transformation begins in the cells with the shortest telomeres.

Our interpretation of these findings is that telomere shortening proceeds within adenomas despite a correlative increase in telomerase activity, a feedback loop insufficient to reverse telomere attrition, likely because differentiating cells lose the capacity of their precursors, stem cells, for telomerase expression. At some point that marks the onset of immortalization, either telomerase re-expression or ALT will take place, and telomere length stabilizes, an indicator of malignant conversion. The precedent findings suggest that gradual telomere shortening (not necessarily a definitive reduction in length but some level of telomere damage) is the trigger for the re-expression of telomerase or ALT and telomere length stabilization.

The signaling pathway that connects progressive telomere shortening or damage to telomerase activation (or ALT) must be different from the mechanism underlying telomerase activation in stem cells. First, in cells entering the differentiation pathway, including progenitor cells, telomere maintenance is impaired compared to stem cells, as suggested by an increased rate of telomere shortening in intestinal adenomas compared to stem cells. This is consistent with a negative impact of exiting the stem compartment and cell differentiation on the ability of telomerase to cope with telomere erosion (or impaired coupling of cell proliferation with differentiation caused by oncogenes). Later, a fundamental change in telomere maintenance could explain telomere stabilization. Until this point, the increased response in telomerase activity is unable to counteract continuous telomere shortening. The dual nature of this process—accelerated telomere shortening and the appearance of a new permanent mechanism of telomerase control that coincides with immortalization—strongly suggests that immortalization is facilitated by the preceding process of telomere shortening and does not occur in the stem cell compartment. Instead, it occurs in a cell engaged in tissue turnover and some degree of differentiation before developing a new mechanism for telomere maintenance distinct from the stem cell mechanism. Both concepts are supported by recent discoveries on telomere reactivation in cancer (to be discussed). First, telomerase expression is controlled differently in malignant versus pre-malignant cells. Second, permanent reactivation of telomerase (or ALT) inherent to cell immortalization emerges simultaneously with other stem cell features and therefore occurs in a non-stem cell population. As stated above, oncogenes’ interference in cell turnover appears to be a constant finding in tumor development. In a former study, we showed that a true stem cell phenotype is extremely rare in cancer [9]. Even tumors that reproduce phenotypic features of cells at early stages of development exhibit premature differentiation [9]. In the intestine, it is well-established that colon cancer-initiating cells are included in the CD133-positive population. By limiting dilution analysis, it was determined that there was one cancer-initiating cell among 263 CD133+ cells, a >200-fold enrichment over the unfractionated population. The CD133- cells did not initiate tumor growth [24]. Prominin was detected throughout the lower half of the intestinal crypt and was expressed beyond the rare stem cell subset sandwiched between Paneth cells. CD133 can be detected in Lgr+ stem cells and in the more numerous transit-amplifying cells. Thus, it is highly likely that colon cancer-initiating cells are early-stage committed precursors rather than stem cells.

Hoffmayer [25] showed that Wnt/β-catenin signaling regulates telomerase expression in the intestinal mucosa and mouse ES cells. They revealed binding sites in the hTERT promoter and transcription start sites for Wnt targets. The complex arrangement of distinct targets of the Wnt/TCF pathway on the hTERT promoter enables a flexible response of telomerase expression in different contexts or the occurrence of mutations at these promoter binding sites. This could explain differences in control in cancer and adenoma, even with minimal promoter changes. Thus, when colon carcinoma cell lines carrying tetracycline-inducible constructs coding for dominant negative versions of TCF1 or TCF4 were subjected to tetracycline treatment, significant downregulation of TCF targets expressed in colon carcinomas were observed, but hTERT transcript levels were similar to levels in tumors lacking the dominant negative constructs. As Ducrest et al. stated, “these results quite strongly argue that TCF does not play a role, direct or indirect, in controlling hTERT expression in colon carcinomas” [26]. In cancer transformation, telomerase re-expression occurs under a new, different type of control compared to in normal stem cells.

New discoveries [27,28] reveal the bewildering complexity of the mechanisms used by cancer cells to support telomerase reactivation, all of which appear to be specific to cancer cells and linked to the emergence of stemness. Cancer-specific hTERT transcription may occur due to hTERT transcription under the control of a mutated hTERT promoter or the wild-type promoter, which is responsible for alterations in the nearby chromatin architecture.

A report from Kim et al. [27] describes a new phenomenon that they named the telomere position effect over long distance (TPE-OLD). Their report explains how telomere length is linked to telomerase expression. In cells with long telomeres, chromatin folding facilitates loop formation, placing the subtelomeric region adjacent to the TERT locus. This is associated with repression of TERT expression. Conversely, telomere looping between the hTERT locus and subtelomeric 5p, which exists in normal cells, is greatly reduced during in vitro aging, leading to abrogation of TERT repression. These researchers point out that the hypothesis that the hTERT gene is not transcribed in normal telomerase-silent cells is likely incorrect. Expression of splice variants takes place but does not result in full-length RNA capable of producing telomerase activity. However, they observed higher transcription in the cells with the shortest telomeres. They also observed higher levels of active telomerase in two human fibroblast strains with short telomeres compared to the same cells with long telomeres. Chromatin looping affects the expression of genes located in the vicinity of the TERT locus, such as the CLPTM1L gene. CLPTM1L mRNA expression was detected in normal passaged BJ human fibroblasts, but its expression increased as telomere erosion progressed. The expression of genes located at intermediate distance between CLPTM1l and the telomere locus did not change. The active chromatin marks H3K4me3 and H3K9 acet were found to be increased across the hTERT promoter of aged cells with short telomeres compared with young cells with long telomeres. The same active transcription marks were detected at the promoters of nearby genes such as CLPTM1L.

Together, these findings suggest that telomere shortening creates permissive conditions that may not be sufficient for the production of active telomerase on their own and require the participation of other factors, such as oncogenes. With this idea in mind, researchers tested the knockdown of p21 (CDKN1A), which had previously been shown to derepress hTERT expression. This resulted in an increased number of transcripts, including later exons 7/8 (coding for critical residues in the reverse transcriptase domain) and exons 15/16, which are responsive for putative active hTERT and total variants, respectively. These variants increased with the knockdown of p21 in old but not young BL cells, but no telomerase activity was detected. This suggests that more drastic alteration or further shortening of telomeres may trigger the reactivation of telomerase.

The role of oncogenes (or epigenetic alterations mediated by oncogenes in concert with genomic changes in the proximity of the telomerase promoter) in the reactivation of TERT expression is exemplified by another mechanism of Tert regulation. This mechanism was revealed by Akincilar et al. [28], who studied samples from CRC patients and found a significant correlation of JunD and β-catenin expression and hTERT transcription. Since JunD co-localizes with CTCF to regulate chromatin compactness, the authors compared RNA-seq and ATAC-seq data between high-JunD and low-JunD CRC cells and normal colon cells, identifying 10 accessible chromatin regions co-enriched with JunD and CTCF that were not present in stem cells. Removal of a distal non-coding region located 140 kb upstream of the WT promoter abrogated hTERT expression, demonstrating the influence of chromatin architecture on hTERT expression. JunD and CTCF were both only required for hTERT expression in cells containing a wild-type promoter, and their deletion abrogated binding of Sp1 to the WT-hTERT promoter. Afterwards, circularized chromosome conformation capture (4C) sequencing identified a 5 kb T-INT2 region (referred to as Tert interacting region 2) that regulates WT-hTERT expression. It was proposed that JunD-CTCF binding relaxes and bends chromatin, facilitating an interaction between T-INT2 and WT-hTERT.

JunD upregulation of Tert in CRC cells also involves the recruitment of epigenetic factors, including CBP/p300, which are known to unwind chromatin in conjunction with other epigenetic modifiers, like CTCF and Sp-1, to form bounds between distinct chromatin regions. In CRC patient samples, a significant correlation was found between JunD and β-catenin expression. Although knockdown of β-catenin with siRNA reduced hTERT expression in HCT116 cells, it did not affect hTERT expression in HT29 cells, which have low basal β-catenin levels. The INT-2 region is operative in CRC cells in which hTERT is activated by oncogenic alterations but is not found in normal stem cells or induced pluripotent cells in which the four factors keep the WT-hTERT promoter active. INT-2 does not participate in the regulation of mutated hTERT promoters, which are regulated by yet another mechanism involving long-range chromatin interactions.

Mutations of the hTERT promoter are common in cancer, with a higher incidence in glioblastoma, melanoma, urothelial, bladder, and thyroid cancers, and they tend to affect older patients. Point mutations of the TERT promoter have been identified in two key positions, C250T and C228T, in 19% of cancers. In general, these are single-base mutations that create binding sites for members of the ETS transcription factor family [29]. These transcription factors (TFs) enhance telomerase activity mediated by dimerization with their family or with other TFs such as NF-κB. Regulation of mutant promoters appear to require long-range chromatin interaction between the mutant promoter and a genomic region located 260 kb upstream. This genomic region, called T-INT1, contains multiple binding motifs for GA-binding protein (GABP) α/β (members of the ETS transcription family). GABPA dimers on the proximal promoter have been shown to interact with GABP dimers located in a region 260 kb upstream of the TSS site of the hTERT gene to make a stable transcriptional hub [30]. The MED12 subunit of the MED complex was identified as a mediator of that interaction. The same report identified other candidate genes for similar long-range interactions that require further validation. T-INT1 appears to regulate only mutant TERT promoters.

## 5. Complementation Between Oncogenic Pathways Suggests Parallel Changes in Reprogramming of Stemness and Telomerase Control

As shown previously, adenomas display a crypt/adenoma TCF-dependent gene expression signature. SOX4, LGR5, AXIN2, cMYC, and this signature, together with tumor cell proliferation, are abrogated in vitro by the inhibition of TCF function through dominant-negative TCF (dnTCF4) expression. Hedgehog (HH)-GLI is another signaling pathway important in colorectal carcinogenesis (CCs). HH-GLI has been shown to be essential to the proliferation and survival of primary colorectal carcinoma cells. In this pathway, signaling is normally triggered by secreted HH ligands, most often by Sonic Hedgehog (SHH), that inactivate the 12-transmembrane protein Patched1 (PTCH1). PTCH1 activity inhibits the function of the seven-transmembrane G-couple receptor-like protein Smoothened (SMOH) Upon PTCH1 inactivation by HH ligands, SMOH is free to signal intracellularly, involving several kinases and leading to the activation of Gli transcription factors. Gli factors have activator and repressor functions. Gli3 3 encodes the strongest repressor. In the absence of HH ligands, GliR is dominant, and Gli1 is not transcribed. Upon SMOH activation, the Gli code is switched so that Gli1 is transcribed and Gl3R is repressed.

In a panel of tumors isolated directly from patients and processed, Varnat et al. [31] detected the Wnt/TCF signature (LGR5, SOX4, Axin2, cMYC, and CD44) in CD133^+^ cells from early, non-metastatic TNM1,2 CCs compared to normal colon cells, whereas this signature was downregulated on metastatic TNM 3,4 CD133^+^ and CD133^−^ cells. Instead, the latter displayed elevated expression of the HH-GLI signature. Metastatic CCs also exhibited higher levels of the Wnt/TCF inhibitors DKK1 and SFRP1 than non-metastatic CCs. Inhibition of a crypt/adenoma signature by dnTCF4 did not decrease the expression of HH-Gli1 pathway activity markers and vice versa. Blockade of endogenous HH-Gli1 repressed Gli1 but did not inhibit Wnt-TCF, suggesting that, in vitro, Wnt-TCF and HH-Gli act in parallel. Analysis of cell lines and primary CCs revealed that expression of the Wnt/TCF signature was maintained in cells cultured in vitro whereas repression of this signature and enhanced expression of the HH-GLI and core ES-like signatures were detected in vivo.

In addition, a stem cell-like signature formed by NANOG, OCT4, SOX2, KLF4, and BMI1, similar to the reprogramming set involved in inducing iPS cells from differentiated cells, was enriched in CD133^+^ versus CD133^−^, especially when comparing metastatic and non-metastatic cases. The stem-like signature was dependent on the HH-GLI pathway but was not affected by blockade of endogenous Wnt/TCF signaling by dnTCF4.

The finding that β-catenin induced GLI1 activity in a GLI-binding site luciferase reporter assay independent of TCF function suggests crosstalk between these pathways. KRAS^v12G^, MEK, and AKT also enhanced GLI activity on GLI–luciferase reporter assays. Exogenous Gli activity was also repressed by PTen and p53. Interestingly, MEK and AKT inhibitors repressed the epithelial–mesenchymal marker SNAIL1 in metastatic CCs only. In addition, β-catenin has been found to upregulate sonic hedgehog (Shh) and Patched (PTCH1), as well as IGF signaling on the hair follicle [32].

Repression of the Wnt-TCF crypt/adenoma signature in patient samples and in xenografts versus in vitro cultures indicated the possibility that Wnt-TCF signaling might be replaced by other signaling pathways during tumor progression, ending in further reprogramming (with immortalization potentially emerging in cells within the same population through a secondary oncogenic pathway). Researchers made use of two established CC cell lines previously used to demonstrate the key role of TCF function in vitro: Ls174T-dnTCF^dox^ and DLD1-dnTCF^dox^. These clones display homogeneous dnTCF4 expression only upon doxycycline addition. Ls174T-dnTCF^dox^ transplanted in vivo displayed TCF target inhibition to the same level as Ls174T-dnTCF^dox^ transplanted in vitro when compared with untreated cell controls, but this did not have an impact on tumor growth: tumors resulting from Ls 174T-dnTCF4^dox^ cells or parental Ls174T cells with or without dox treatment showed similar growth curves and tumor appearance. However, the same level of inhibition in vitro led to complete growth arrest. This lack of tumor growth arrest despite the inhibition of several targets of Wnt-TCF parallels the results described above [26] and suggests a critical change in tumor growth control probably associated with separate control of telomerase expression. However, results for DLD1-dnTCF4^dox^ were different. Treatment with dox repressed TCF targets and tumor growth both in vivo and in vitro, possibly indicating that telomerase expression control had not yet been transferred [31].

Further research suggests again that over the course of cancer progression, the initial oncogenic pathways may intersect or overlap with new cancer-driving pathways. This process might entail a secondary reprogramming event within an established cancer stem cell or within another cell from the tumor population. Findings by Asciutti et al. [33] show the superimposition of distinct oncogenic pathways on carcinogenesis initiated by the Wnt/TCF pathway. They studied three gastric carcinomas: AGS that harbors a β-catenin-activating mutation that prevents its proteasome degradation and MKN-28 and MKN-74, both of which contain inactivating mutations of APC. Downregulation of TCF signaling by dominant negative TCF4 (dnTCF4) led to reduced expression of TCF targets, including c-Myc, BMP-4, Axin-2, and Lef-1, in all three tumors, as well as inhibition of tumor cell proliferation in AGS. No inhibition of proliferation was observed in the other two cell lines despite a comparable response to dnTCF4 regarding the downregulation of TCF targets, including c-Myc inhibition, and a correlated increase in p21 levels. Following overnight serum starvation, ERK and AKT phosphorylation were detected in the AGS and MKN-28 tumor lines, while MKN-78 showed minimal if any AKT phosphorylation and very low levels of ERK activation. AGS tumor growth could be inhibited by either dnTCF4 or c-Myc shRNA, although neither affected MKN-28 tumor growth. Exposure to an MEK inhibitor or a PI3K inhibitor led to marked inhibition of MKN-28 tumor proliferation and a slight reduction in AGS proliferation.

## 6. Epithelial–Mesenchymal Transition Appears to Entail a Secondary Stemness Reprogramming Event and a New Change in the Control of Telomerase Reactivation

The superimposition of secondary oncogenic pathways during tumor progression is associated with some ominous signs, such as invasion and metastasis. The onset of metastasis is usually preceded by the invasion of tumor cells into the surrounding soft tissues. The capacity for invasion is rooted in a physiological mechanism that facilitates remodeling during embryogenesis and is later used frequently by cancer cells: epithelial–mesenchymal transition (EMT). A study by Medici et al. [34] on colon cancer and melanoma cell lines demonstrated intricate coordination between the Wnt and TGF-β pathways to facilitate the EMT. Initially, TGFβ1 and TGFβ2 induction of Snail and Slug resulted in decreased levels of E-cadherin and the subsequent release of cytosolic β-catenin. Then, β-catenin-TCF4 upregulated TGF-β3, which promoted the synthesis of LEF-1. As Snail and Slug are transcription repressors, their induction of TGFβ3 is indirect and mediated by β-catenin-TCF4 binding to the TGF-β3 promoter.

Loss of E-cadherin was accompanied by morphological changes characteristic of EMT. However, DN-LEF-1 prevented invasion associated with EMT. Enhanced transcription of TGFβ3 results in the upregulation of LEF-1 which, after binding of cytoplasmic β-catenin, can enforce the acquisition of the mesenchymal phenotype and gene changes associated with EMT: increased vimentin, fibronectin, α-SMA and α-actinin expression.

The authors indicated the possible involvement of the MAPK pathway in the TGFβ1/2 upregulation of the E-cadherin repressors Snail and Slug, as well as a possible role of GSK3β, an important node in the bifurcation of MAPK and other signaling pathways. TGFβ1, TGFβ2, and TGFβ3 all signal through PI3K (and downstream target AKT), which induces phosphorylation and inactivation of GSK3β, thereby releasing β-catenin from its destruction complex and creating a surplus of β-catenin that can associate with the LEF-1 upregulated by TGFβ3 signaling.

The report by Medici et al. does not address the emergence of stem cell traits or the potential role of telomerase in EMT. This subject was addressed in a model of gastric cancer by Liu et al. [35]. Wnt-TCF supports the self-renewal of gastric mucosa, and its deregulation can initiate gastric cancer, but EMT appears to require the participation of hTERT in addition to the other factors described above. Liu et al. infected the GC cell line BGC-823 (and two more gastric cancer cell lines) with an hTERT retroviral vector and detected increased levels of the mesenchymal markers vimentin and Snail and reduced E-cadherin. These conditions resulted in significantly increased invasiveness of the hTERT-BGC-823 cells compared with control pBabe-BGC-823 cells. Conversely, hTERT inhibition resulted in the loss of these mesenchymal markers and diminished invasiveness. hTERT inhibition by siRNA substantially abolished TGFβ1-stimulated Snail and vimentin induction and, conversely, the hTERT-overexpressing cells exhibited much higher levels of Snail and vimentin than the control cells. hTERT and TGFβ1 affected β-catenin expression, with the overexpression of these factors leading to higher β-catenin levels and their inhibition correlating with a slight but consistent reduction in β-catenin. Increased expression of Snail and vimentin and activation of the TCF/LEF reporter were detected in BGC-823 cells transfected with a dominant negative hTERT (mutation D869A) that loses a catalytic function or with C-terminal tagged hTERT that exhibits telomerase activity but is unable to elongate telomeres.

After the injection of hTERT-BGC-823 cells or pBabe-BGC-823 control cells into the tail veins of nude mice, metastatic colonies were found in the lungs, but those formed by the hTERT-BGC-823 cells were larger and more numerous. The hTERT-induced EMT was accompanied by the appearance of stem cell features, such as dramatic enhancement of monosphere formation and the overexpression of stem cell markers like OCT-4 and CD44, an established gastric CSC marker [35]. These findings and the aforementioned study by Varnat et al. suggest a need for simultaneous reprogramming (as shown by the onset of stem cell traits) and the presence of EMT inducers, such as Snail and vimentin.

TGF-β1-mediated upregulation of β-catenin by hTERT and the synergistic effect of TGF-β1 and hTERT in EMT induction demonstrate a positive feedback loop between these factors that may accelerate the induction of EMT and the acquisition of stemness traits. It is also possible that during cancer progression, the interaction of hTERT with new partners leading to increased hTERT expression might induce further reprogramming events that could accelerate and worsen the course of the disease.

## 7. Id1 and Epithelial–Mesenchymal Transition

Ids are members of the helix–loop–helix (HLH) group of proteins, which can form heterodimers with basic helix–loop–helix (bHLH) proteins. Lacking the basic domain necessary for DNA binding, Ids act as dominant negative transcription factors. For instance, in hematopoiesis, Id1 may preserve the undifferentiated state by antagonizing a differentiation step induced by E2A [36]. This property is likely involved in directing reversal of epithelial differentiation towards mesenchymal differentiation.

When bone marrow cells were transduced with an Id1 expressing MSCV retrovirus, they could divide for over 1 year in the presence of a stem cell factor. Transplantation of cultured cells to mice induced a myeloproliferative-like disease. However, secondary recipients did not develop leukemia [9], suggesting that prevention of differentiation by Id1 does not induce transformation.

The results afforded by in vivo experiments are somewhat different. One in vivo study on Id1 took advantage of a murine mammary epithelial cell line, SCp2, which can differentiate in vitro, recapitulating the main aspects of development and lactation, including proliferation and mammary invasion during late pregnancy, as reflected by growth arrest and the formation of alveolar structures and milk proteins when the serum is removed and cells are placed in contact with basement membrane components and given lactogenic hormones (insulin, prolactin, and hydrocortisone). Upon constitutive Id1 expression, these cells slowly invade the basement membrane and express a 120 kd metalloproteinase (MMP), mimicking the capacity of normal mammary cells to secrete MMPs that degrade the ECM during involution. However, despite constitutive Id1 expression, SCp2 cells do not grow in soft agar or form tumors in nude mice. SCp2 cells transfected with the murine Id-1 cDNA driven by the mouse mammary tumor virus promoter (Cp2-Id-1 cells) formed alveolar structures but later (10 days) detached and migrated through the surrounding parenchyma. Among nine human breast tumors examined, only Id-1-expressing cells also expressed the 120 kDa gelatinase. A complete epithelial–mesenchymal transition was not observed, as they still retained some epithelial markers [37].

Expression of activated Ras in mammary epithelia of mice leads to the formation of senescent foci containing disorganized epithelial cells. Mice that concurrently harbor a deletion of the p19, p53, or p21 tumor suppressor rapidly develop mammary tumors, suggesting that these tumor suppressors prevent tumorigenesis by inducing senescence. However, ectopic expression of Id1 co-expressed with activated Ras leads to rapid tumor development, which indicates the capacity of Id1 to antagonize apoptosis induced by the tumor suppressor axis of p19, p53, and p21. In the same study, conditional activation of Hras^ᴧ^V12 in conjunction with constitutional activation of Id1 led to tumor formation, but doxycycline inactivation of the Hras^ᴧ^V12 promoter resulted in complete tumor regression within 40 days. This suggests that Id1 alone, even if constitutively expressed, cannot give rise to tumor development. However, as the same study shows, it is true that overexpression of Id1 in the intestine leads to the development of adenomas, albeit with long latency and low penetrance. To our knowledge, there is only one example of a tumor (leukemia) induced by Id1 alone. Its overexpression in the hematopoietic compartment leads to massive apoptosis, disruption of differentiation, and leukemia [38]. The induction of massive apoptosis by Id1 is surprising as it seems to contradict the anti-apoptotic function usually performed by Id1. The transgenic expression of Id1 in thymocytes was found to change the normal distribution of the thymocyte population at the DN and later stages in a way that is highly compatible with a hematopoietic developmental block, which is a frequent cause of leukemia. This suggests a totally different mechanism of tumorigenesis that must be imputed to the developmental block [39].

In summary, Id1 does not meet the classical definition of an oncogene. No Id1 mutations have been found, although numerous reports have linked Id1 to the induction of invasion and metastasis in human cancer through promotion of the epithelial–mesenchymal transition (EMT). Two characteristic features of epithelial cells undergoing EMT are the adoption of a mesenchymal morphology and the simultaneous expression of stem cell properties. This demonstrates the possibility of spontaneous reprogramming of committed cells to neoplastic stem cells. However, most information on EMT has been obtained with models involving the ectopic expression of Id1 in immortalized cell lines, although some have involved committed cells. The main final downstream effectors of Id1 in EMT include negative regulators of E-cadherin, such as Snail, Twist, fibronectin, and vimentin, although various additional targets participate in this process in distinct tissues. Some examples are provided below.

## 8. Breast

Mani et al. induced EMT via the ectopic expression of Snail or Twist in cells isolated from non-tumorigenic, immortalized mammary epithelial cells (HMLEs) sorted into enriched mammary stem cells (CD44^hi^ CD24^lo^ and committed CD44^lo^ CD24^hi^). The mesenchymal-like cells generated by EMT acquired a CD44^hi^ CD24^lo^ expression pattern, precisely the same phenotype that has been ascribed to neoplastic mammary stem cells. Similarly, the induction of EMT in HMLEs by exposure to TGF-β1 (an inducer of Id-1) resulted in the appearance of cells with a mesenchymal appearance and the acquisition of the CD44^hi^ CD24^lo^ phenotype [40]. In the words of these authors, “In principle, the behaviour of the HMLE human mammary epithelial cells might be attributable to the introduced genes that were used previously to immortalize these cells, specifically hTERT, which encodes the catalytic subunit of human telomerase holoenzyme, as well as the SV40 early region. For example, these genes might facilitate the reprogramming of the HMLE cells initiated by EMT-inducing transcription factors such as Snail or Twist. However, we observe the exact same responses to Snail or Twist in non-immortalized primary mammary epithelial cells, which indicates that hTERT and SV40 antigens appear to have no effect on the induction of the EMT. In summary, the experiments indicate that committed cells are reprogrammed to stem cells when subjected to EMT.”

Other experiments have revealed the wide diversity of downstream targets that may be involved in Id1-induced EMT. Gumireddy et al. [41], who also used breast cancer cell lines and clinical samples, showed that Id1 interacts with TFAP2A, which binds to the S100A9 promoter, enhancing its activity. A consequence of this activation is the suppression of RhoC. Therefore, by binding TFAP2A, Id1 downregulates RhoC. RhoC appears to be the main protein responsible for the invasion and metastasis of breast cancer, and its downregulation is suppressed the migratory and invasive phenotype induced by Id1.

Other experiments have indicated that the PI3K/AKT pathway is the backbone of EMT. In the breast cancer cell line MCF7, Lee et al. [42] showed that Id-1 activated the Akt pathway by inhibiting PTEN transcription through the downregulation of p53. Akt was phosphorylated at Ser473 and glycogen synthase kinase at Ser9 with stabilization and nuclear localization of β-catenin, TCF/LEF, and cyclin D1, Akt-mediated p27 phosphorylation at Thr157, and translocation to the cytoplasm. Fong et al. reported that infection of a non-invasive breast cancer cell line with Id-1 rendered it invasive [43].

A lung cancer study confirmed that Id1 induction was mainly mediated by Akt activation. Id1-overexpressing H460 lung cancer cells demonstrated elevated expression of cyclin D1 and CDK4 and decreased expression of CDK2, cdc2, and cyclin B1. The expression of phospho-p38MAPK (the active form of p38MAPK) was increased in Id1-overexpressing H460 cells and repressed in Id1-knockdown H520 cells. However, when Id1-overexpressing H460 cells were treated with SB203580, an inhibitor of p38MAPK, or wortmannin, an inhibitor of PI3K/Akt, they found that only wortmannin had a significant effect on suppressing cell proliferation, indicating that Akt was the main effector of Id1 expression. The activity of JNK, ERK, and STAT3 was also analyzed [44]. Activation induced by Id-1 may extend a step further along the PI3K pathway to also affect NF-κB expression. Li [45] showed that in esophageal cancer cells, stable ectopic expression of Id-1 induced the expression of pAKT, glycogen synthase kinase 3β, and inhibitor of kappa B, as well as increased nuclear translocation of NF-κB subunit p65 and NF-κB binding activity. These effects were antagonized by treatment with the PI3K inhibitor LY294002. This experiment revealed the involvement of another downstream target of the pathway, NF-κB, which appears to be responsible for protection against apoptosis in cancer cells. Both the PI3K inhibitor LY294002 and the NF-κB inhibitor Bay 11-7082 increased the sensitivity of Id1-overexpressing esophageal cancer cells to TNF-α-induced apoptosis.

The same conclusion was derived from another study of prostate cancer cells. Id-1 induced nuclear translocation of NF-κB and the upregulation of its downstream targets Bcl-xL and ICAM-1, which was accompanied by increased resistance to apoptosis induced by TNFα through the inactivation of Bax and caspase 3 [46].

## 9. Liver

The direct origin of cancer from primary hepatocytes has been described in association with or preceding EMT. Cirrhotic livers provide a fertile soil for cancer development, and some observations implicate fibroblastoid cells derived from hepatocytes as the cells of origin of hepatocarcinoma. TGF-β, which is increased in chronic inflammation of the liver, can promote this phenotypic change. Primary mouse hepatocytes have been observed to lose their epithelial phenotype in response to TGF-β accompanied by the loss of E-cadherin and Snail upregulation. In the liver, EMT appears to be a complex process requiring inputs from several signaling pathways. In primary murine hepatocytes, EMT was associated with the focal adhesion kinase (FAK)-Src-dependent activation of the PI3K/Akt and Erk1/2 pathways. In human hepatocarcinoma, EMT appears to be associated with metastatic and highly invasive tumors. E-cadherin was reported to be strongly expressed upon cell-to-cell contact in samples from a group of non-metastatic HCC patients. Instead, reduced expression of E-cadherin and loss of cell-to-cell contact were associated with nuclear translocation of β-catenin and predominated in the group of HCC patients with metastasis [47].

In cervical cancer, the T-box transcription factor 3 (TBX3) promotes EMT, and its expression correlates with Id1 mRNA and protein levels, although it has not yet been confirmed whether TBX3 regulates Id1 directly. Silencing TBX3 reduced cell migration and invasion. However, TBX3 may also act through its ability to downregulate PTEN and suppress the p19-p53 pathway [48]. In another study on cervical cancer, Id1-induced activation of the PI3K pathway could be followed to downstream target NFkBp65 [49].

Caveolin-1 is essential to the role of Id-1 in inducing EMT and cell survival in prostate cancer cells. Id1 and caveolin alone can induce EMT, but they have a synergistic effect. In prostate cells, Id1 induced Akt (mediator of the PI3K pathway) activation by promoting binding between Cav-1 and PP2A. Cav-1 regulates signaling of membrane proteins, such as epidermal growth factor receptor, Src, and H-ras, through direct physical interaction. Although Id1 and Cav-1 are each able to increase Akt phosphorylation alone, they can act synergistically. Id1 promotes the inhibitory effect of Cav1 on PP2A, but this does not result in a decrease in PP2A levels. Id1 does not bind PP2A. Thus, it seems that Id1 promotes binding between Cav-1 and PP2A, which may result in suppression of PP2A activity [50].

## 10. Glioblastoma

As first described in breast cancer, TGF-b has been shown to increase the CD44high cell population through Id1. Initiating tumor ability is contained in the CD44high/Id1high compartment. Treatment of human GBM with a TGF-β inhibitor resulted in fewer tumors upon transplantation to mice transplanted to mice [51].

Other experiments in non-transformed p16-p19-deficient astrocytes have revealed how the context can influence backbone signaling pathways; Id1-mediated suppression of Cullin3 conferred stem cell-like features and tumorigenicity to Ink4a/Arf^−/−^ astrocytes. The authors demonstrated that ID1-mediated suppression of Cullin3 may activate SHH and Wnt signaling by stabilizing the GLI2 and DVL2 proteins, respectively. However, qRT-PCR analysis revealed that Dvl2 and Gli2 mRNA levels were not significantly changed by ID1 overexpression, which indicates that ID1 regulates DVL2 and GLI2 expression at a post-transcriptional level by suppressing Cullin3 expression [52]. This phenomenon also occurs in tumorigenesis: the initial extracellular inducers of a tumorigenic signaling pathway cease to be necessary for stimulation of the pathway, which becomes autonomous, that is, dependent only on the intracellular downstream targets. The ID1high/Cullin low signature correlates with poor patient prognosis.

Superimposition of the Wnt and Shh signaling pathways has also been demonstrated in the colon cancer line HCT116. The combined inhibition of the Wnt pathway with FH535 and the inhibition of the Shh pathway with HPI-1 resulted in greater inhibition than the effect of either inhibitor alone. Concomitantly, there were significant reductions in c-myc expression and the c-myc downstream target PLAC8. Taken together, these data demonstrated that Id1 maintains the stemness of CRC cells via the Id1-c-Myc-PLAC8 axis by activating the Wnt/β-catenin and Shh signaling pathways [53].

A few reports have uncovered the participation of EGFR and MAPK in Id1 expression. In non-small-cell lung (NSCL) cancer, Pillai et al. [54] reported that signaling through the nicotine acetylcholine receptor or the EGF receptor upregulated Id1 in an Scr-dependent manner. Nicotine induced the expression of the mesenchymal markers fibronectin and vimentin by downregulating ZBP-89, a zinc-finger repressor protein. The ectopic expression of a mutant Ras also resulted in increased Id1 levels.

Despite the above reports, it is important to emphasize that EMT occurs as a physiological phenomenon in wound healing. EMT takes place in the early phase of wound healing, as demonstrated by a change in keratinocyte morphology, which shifts to that of interstitial cells with the capacity to migrate, and the typical molecular expression pattern of EMT—the downregulation of E-cadherin and the overexpression of vimentin and N-cadherin. This molecular expression pattern was amplified in the keratinocyte cell line HaCat by the addition of TGF-β1. Exogenous TGF-β1 has also been shown to accelerate cutaneous wound healing, and Foxo3a was shown to revert these changes. Overexpression of Foxo3a suppressed the TGF-β1-induced EMT and associated β-catenin activation. β-catenin, a key molecule of the Wnt pathway, was increased in the cytoplasm and nucleus of HaCat cells by silencing Foxo3a expression; conversely, the expression of β-catenin was decreased by infecting HaCat cells with lenti-flag-Foxo3a [55]. Here, a new player, FOXO, enters the game. There are reports showing that Foxo3a can suppress EMT in prostate cancer cells by inhibiting the Wnt pathway. Foxo3a inhibits β-catenin through both direct binding and inducing miR-34 expression. β-catenin was shown to participate in the Foxo3a-mediated suppression of EMT. In fact, decreased E-cadherin and increased N-cadherin and fibronectin levels induced by Foxo3a KD and other features of EMT were reversed by silencing β-catenin expression [56].

**Figure 1:**
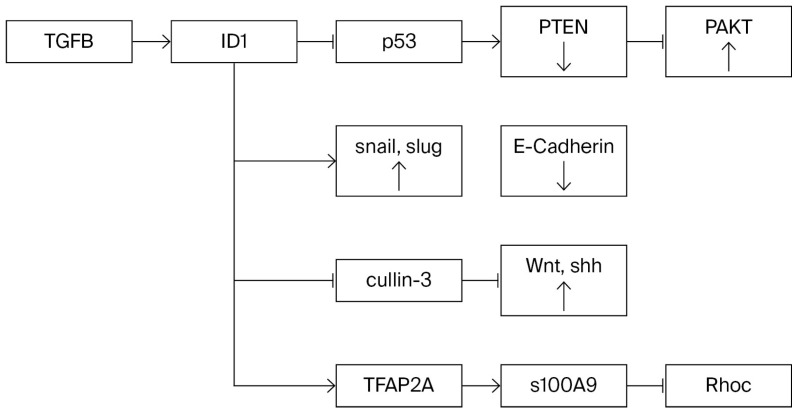
Some circuits involved in increased cell migration, invasion, and metastasis.

Evidently, EMT is a very complex process, about which we have only fragmentary information, as illustrated in Figure 1.

The above observations illustrate the crucial role of crosstalk between the PI3K and Wnt/Shh oncogenic pathways. Some of the reports gathered describe the activation of a downstream molecule in these and other pathways, NF-κB, which is involved in stemness and invasiveness. However, the common occurrence of stemness could be explained if oncogenic signaling extends to other downstream targets of the PI3K pathway, such as the FOXO family, particularly Foxo3a, which are known to regulate stemness.

## 11. FOXO and NF-κB Interplay (Figure 2 and Figure 3)

**Figure 2:**
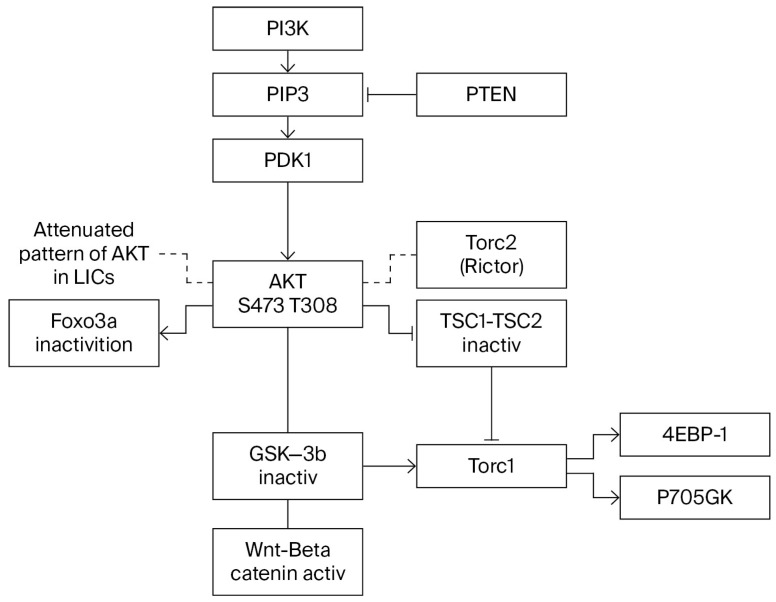
P13K/AKT signaling.

The family of Forkhead box transcription factors consists of 19 subclasses of Fox genes. These genes are involved in stemness, cell survival, redox homeostasis, and stress resistance. In the absence of growth factor or insulin stimulation, Foxos reside in the nucleus, where they activate transcription of cell cycle inhibitors such as p130, p27, p57, and p21 and proapoptotic genes such as TRAIL, FasL, and Bim. Foxo inactivation by PI3K/AKT signaling leads to its nuclear exclusion and enhanced cell proliferation, survival, and stress sensitivity [57]. Loss of Foxo in the hematopoietic compartment results in an increase in HSCs in the cell cycle accompanied by the exit of CD34^−^ LSK cells and concomitant decreases in p27 and p57. Stem cell quiescence depends on these cyclin-dependent kinase inhibitors (CKIs). Ablation of CKIs leads to stem cell exhaustion [58]. The transition from HSCs to myeloid progenitors is accompanied by a 100-fold increase in ROS. This change appears to be regulated by a transcription program that is independent of Foxo. The high ROS level that is appropriate for the functions of short-lived myeloid cells is incompatible with HSC homeostasis [59]. Akt is the main kinase that regulates Foxos (Figure 2), although other kinases, such as glucocorticoid inducible kinase (SGK), casein kinase (CK1), dual thyrosine phosphorylated regulated kinase 1 (DYRK1), and IKK, may phosphorylate Foxos in specific residues and target many other protein substrates including GSK3β, p21, and p27. Phosphorylation of Foxos by JNK appears to be counterregulatory with respect to Akt [59,60].

Studies on the role of the PI3K pathway in tumorigenesis have revealed two apparently reversible functions of FOXOs associated with the activation and inactivation of AKT. The inactivation of Foxo3a, which is always accompanied by cytoplasmic translocation of Foxo3a, results in cell proliferation–differentiation. However, despite AKT signaling, some cells show nuclear localization of Foxo3a and a pattern of attenuated AKT (Figure 2). This reverse nuclear translocation opposite to the expected cytoplasmic translocation normally induced by phosphorylated AKT appears to be essential for the emergence of the cancer stem cell and the maintenance of leukemia-initiating cells, whereas the suppression of Foxo3a appears to be essential to maintaining survival and proliferation. This double shuttling of Foxo3a is observed in AML-AF9 leukemia, where leukemia-initiating cells (LICs) have the GMP phenotype but, unlike the bulk tumor cells, display an attenuated pattern of AKT activation. Sykes et al. [60,61] activated AKT in HSCs by Pten deletion or myr-AKT constructs transduced to bone marrow cells, using the stem cell virus MSCV. Invariably, Pten deletion led to HSC mobilization and HSC exhaustion, followed by tumorigenesis. When injected into irradiated syngeneic recipients, BM cells transduced with activated AKT (MSCV-IRES-GFP-myr construct) induced a myeloproliferative disease (MPD) that progressed to either AML or ALL. Despite the MPD, the proportion of GFP^+^ LSK cells were decreased compared to a control, an outcome consistent with HSC mobilization and exhaustion of the HSC compartment. Paradoxically, Sykes et al. found that the same AKT construct induced suppression of growth in a model of AML-AF9 leukemia. The suppression of growth was associated with myeloid maturation and myeloid maturation-related cell death. Pten deletion activates AKT and induces self-renewal and exhaustion of HSCs (likely accompanied by the exit of the HSC compartment). In the AML-AF9 leukemia model, the expression of phosphorylated AKT followed by HSC exhaustion was accompanied by a conspicuous increase in myeloid maturation and myeloid maturation-related cell death (the myeloid marker CD11b was higher in bone marrow cells injected with the stem cell virus MSCV (MSCV.IRES-GFP-myr-Akt)). However, maturation-related death induced by myr-Akt occurred in the presence of rapamycin, suggesting that Akt utilizes pathways other than mTOR for activation of myeloid maturation [60]. HSC exhaustion and differentiation induced by Foxo3a inactivation was demonstrated in competitive repopulation assays. In addition to HSCs, this has been observed in other tissue stem cells. For instance, Foxo1 and Foxo 3 knockdown led to differentiation of intestinal stem cells into goblet cells and Paneth cells. Foxo 3 levels are higher in neural stem cells (NSCs) undergoing self-renewal, and its ablation led to a decrease in the NSC population accompanied by an increase in progenitors and the exhaustion of the NSC pool [58,59]. A parallelism of the effects of different targets of the PI3K pathway is well known, but a subtle difference may be revealed here: pAKT phosphorylates and inactivates different substrates, such as TSC2 and GSK3-β. Interestingly, pAkt Ser473 is dispensable for TSC2 (which activates mTORC1) and GSK3-β inactivation but is required for Foxo3a inactivation (Figure 2). Thus, myeloid maturation and cell death might be attributed to Foxo3a inactivation. Growth inhibition in this system must be attributed entirely to maturation-related cell death since pAKT only reduces the tumor burden and induces apoptosis of the bulk of tumor cells and does not affect leukemia-initiating cells (LICs). However, in AML-AF9 leukemia, LICs have the GMP phenotype but differ from GMPs by a pattern of attenuated Akt activity (reduced pAktThr308 and reduced pAkt Ser473), which is identical to that of normal HSCs and seems essential to maintaining the capacity for self-renewal. In summary, AKT activation induced by MSCV virus improved leukemia by reducing the tumor burden but did not have any effect on AkT in active stem cells. Since HSCs were pushed into cycle and engaged tissue turnover through Foxo3a inactivation, the attenuated pattern of AKT expression that protects stem cells from exhaustion must be acquired after cells have been pushed out of the HSC stage by Foxo inactivation, as indicated by their GMP phenotype. The reversal of the AKT pattern associated with active Foxo3a must be concomitant with the triggering of immortalization and cancer transformation through an unknown signaling pathway. JNK was shown to be activated in response to Foxo deactivation, but its activation resulted in a further increase in maturation-related apoptosis [60,61].

In summary, emergence of LICs exhibiting a GMP phenotype and attenuated pAKT activity must be linked specifically to the transformation process as it seems to entail two contradictory functions of Foxo and suggest that transformation occurred in a differentiated cell. Interestingly, some Foxo family members, such as FOXF2, can regulate stemness differentially in basal and luminal-like breast cells [61,62].

A previous report validated these findings and unveiled new aspects of the issue [62,63]. BCR-ABL activates Akt signaling that suppress FOXO transcription factors, chiefly Foxo3a in the hematopoietic system. It was shown that Foxo3a plays an essential role in the maintenance of CML LICs. Nuclear localization of Foxo3a and decreased Akt phosphorylation are enriched in the LIC population, and as observed by Sykes et al. in an AML-AF9 model, low Akt phosphorylation in LICs correlates with mTORC1 inactivation. To prove the role of Foxo3a in the maintenance of leukemia-initiating cells (LICs), Naka et al. infected immature bone marrow cells from Foxo3a^+/+^ and Foxo3a^−/−^ mice with a retrovirus carrying MSCV-BCR-ABL-IRES-GFP. The infected cells were transplanted into syngeneic recipients. Both recipient groups developed CML-like MPD. Thus, Foxo knockdown, similar to Foxo inactivation is permissive for tumor generation. However, the in vitro colony-forming ability of second-BMT LICs was decreased by the loss of Foxo3a. In recipients of a third BMT, mild CML-like disease developed within one month, but Foxo3a deficiency prevented the propagation of CML cells in the peripheral blood and spleen. Foxo3a+LICs also developed ALL and CML, which was not observed in recipients of Foxo3a−. Thus, Foxo3a (nuclear) is essential for the long-term maintenance of leukemia-initiating potential, whereas Foxo3a inactivation is an early step in the initiation of leukemia. An important additional finding was that TGF-β_1_ signaling controls Akt activation in the nuclei of KLS^+^ cells but not in the nuclei of KLS^−^ (non-LICs), as indicated by the restriction of Smad2/3 phosphorylation to the nuclei of KLS^+^ cells. In view of the above discussion on the role of TGFβ in epithelial–mesenchymal transition, this finding raises questions regarding the involvement of EMT in these leukemia models. Moreover, the role of telomerase re-expression must be investigated, as suggested by above-mentioned experiment by Liu et al. [35], in which hTERT inhibition by siRNA substantially reduced TGFβ1-stimulated Snail and vimentin induction and, conversely, hTERT-overexpressing cells exhibited much higher levels of Snail and vimentin than control cells. Other outcomes of Foxo inactivation that are not usually considered, such as the non-physiological type of myeloid differentiation induced by Foxo deactivation [59,60], may assist in the promotion of tumorigenesis as interference with hematopoiesis may be a cause for tumor formation on its own, as numerous examples of hematopoietic developmental blocks indicate.

**Figure 3:**
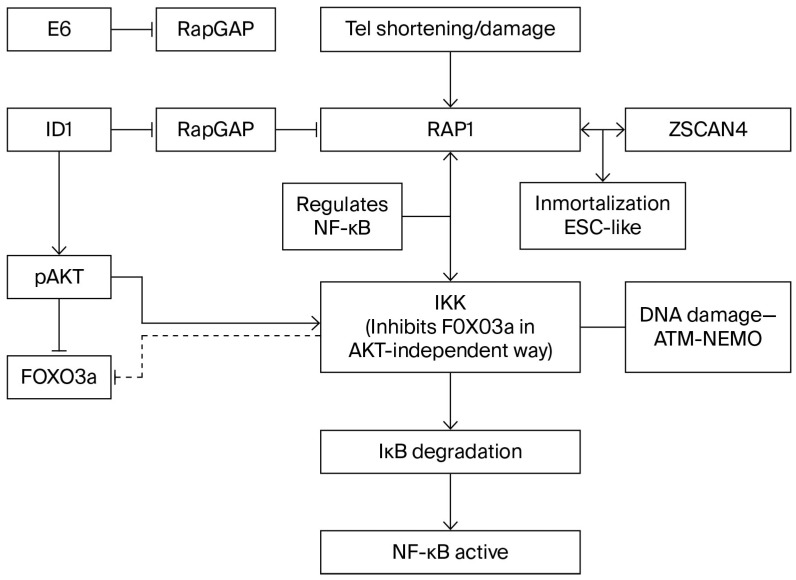
Interaction RAP-Z scan4-telomere damage.

IκB Kinase (IKK_β_, or IKK_α_ to a lesser degree) may induce inactivation and cytoplasmic translocation of Foxo3a independent of pAKT at Ser 644 [63,64] (Figure 3), and both pAKT and IKK-induced nuclear inactivation of Foxo3a promote cell proliferation and resistance to apoptosis. Therefore, it is believed that these molecular changes have an important role in the promotion of tumorigenesis. However, to complicate matters, the correlation between positive pAKT and IKK and the cytoplasmic localization of Foxo3a may be present in normal tissues in addition to tumors [64,65]. Nevertheless, the contribution of Foxo3a to the leukemic transformation is indisputable, as demonstrated by impaired leukemogenesis in Rictor-deleted mice [9] that cannot phosphorylate Akt Ser473, as illustrated in Figure 2, and are therefore unable to inactivate Foxo, suggesting that Foxo’s role in tumorigenesis requires both inactivation in the initial phase within progenitors and activation in LICs.

## 12. NF-κB Transcription Factors and Oncogenesis (Figure 3)

The NF-κB family of transcription factors plays a pivotal role in a multitude of processes and properties, such as inflammation, cell proliferation, survival, adaptive and innate immunity, EMT, and stemness. Here, we discuss its role in cancer as one of the final downstream targets of the PI3K pathway.

The NF-κB family consists of five members: RelA/p65, RelB, c-Rel, NFκB1/p50, and NF-κB2/p52. P50 and p52 lack a transactivation domain and are generated by processing from p105 and p100, respectively.

NF-κappaB signaling is primarily triggered by the IKK complex (IkK_α_, IKK_β_, and IKK_γ_/Nemo), which phosphorylates inactive IκB proteins located in the cytosol, resulting in the nuclear translocation of NF-κB factors and the release and degradation of IκB proteins (Figure 3). Signal transmission to NF-κB transcription targets is channeled according to two different routes, designated as the canonical and alternate pathways.

In the canonical pathway, the IKK_β_ subunit directly phosphorylates NF-κB-associated I_κ_B_α_ (mainly the p50/p65/RelA, which is the most abundant dimer), leading to I_κ_B_α_ degradation and p50/p65 translocation to the nucleus, where it regulates the transcription of target genes.

The alternate pathway relies on IKK_α_ homodimer activation in a manner dependent on NF-κB-inducing kinase (NIK). Activated IKK_α_ phosphorylates p100, yielding p52. Then, the p52: RelB dimer translocates into the nucleus to activate its target genes. Numerous translational modifications modulate the activity of IKK and I_κ_B proteins. The canonical pathway can be activated by a plethora of stimuli, antigen/MHC, cytokines, LPS, IL-1β, and TNF-α. Activators of the non-canonical pathway include the TNFR superfamily, CD40 B-cell activating factor (BAFF), and TNF-like weak inducer of apoptosis (TWEAK). Both pathways can interact at several levels [65,66].

Evidently, NF-κB activation and Foxo3a deactivation are two possible outcomes of IKK activation that can contribute to promoting tumorigenesis.

## 13. NF-κB Can Be Directly Activated Through DNA Damage Signaling

Induction of DNA damage has revealed the existence of an ATM-NEMO-IKK signaling pathway (Figure 3). Grosjean-Raillard et al. [66,67] chose four AML cell lines to study DNA damage-induced NF-κB activation. Increased reactive oxygen species in a KG1 AML cell line that did not exhibit NF-κB activation resulted in phosphorylated ATM and phosphorylation of the IKK complex. It was shown that a complex of ATM and NEMO exited the nucleus to phosphorylate cytosolic IKK, and p65 was translocated to the nucleus. Conversely, ATM inhibition abolished IKK phosphorylation, which was followed by apoptosis. The other three AML cell lines displayed constitutive NF-κB activation, and one of them, P39, also exhibited strong constitutive ATM phosphorylation. In this latter line, ATM inhibition abolished IKK phosphorylation and p65 nuclear translocation. Next, these authors compared ATM-NEMO-IKK signaling between CD34+ myeloblasts (hematopoietic progenitors) from healthy controls and patients with high-risk myelodysplastic syndrome (MDS) or acute myeloid leukemia (AML) (high-risk MDS differs from low-risk MDS in that apoptosis is widespread in the latter and very rare in the former). Less than 1% of myeloblasts from healthy donors stained positive for phosphorylated ATM, whereas a substantial fraction of myeloblasts from either high-risk MDS or AML patients exhibited positive pATM staining. In both AML and MDS myeloblasts, pATM inhibition caused inhibition of NF-κB (loss of nuclear p65), coupled to the inactivation of the IKK complex and the redistribution of NEMO from the nucleus to the cytoplasm.

NFκappaB is constitutively activated in most primary AML samples and in high-risk myelodysplastic syndrome (MDS) but is not activated in normal HSCs [67,68,69]. IKK can be phosphorylated and activated by signaling through the PI3K/AKT or ERK/MAPK pathway, which leads to Foxo3a inactivation and phosphorylation at S644 (and potentially NF-κB activation). Foxo3a can also be phosphorylated by Akt onT32, S253, and S315 and by ERK on S294, S344, and S425. Each of these phosphorylation events has been reported to stimulate FOXO3a nuclear exclusion, ubiquitination, and proteasomal degradation.

Reversion of signaling by inhibition of PI3K or AKT has been shown to result in the redistribution of Foxo3a from the cytoplasm to the nucleus in numerous models, but this was not observed in a study [69] involving primary AML human samples and a leukemic cell line. In a substantial fraction of the samples, Foxo3a was excluded from the nucleus (nuclear localization was detected in only 7.5% of AML samples) despite Akt phosphorylation on S473 and thus phosphorylation of Foxo3a on T32 and S253. However, treatment with IC87114, which terminated these phosphorylation events, did not result in Foxo nuclear translocation. Similarly, the suppression of phosphorylation at ERK1/2 by a specific MEK inhibitor failed to induce Foxo nuclear translocation. In contrast, the anti-NEMO peptide that specifically inhibits IKK activity was found to induce Foxo3a nuclear localization in leukemic cells. Furthermore, an IKK-insensitive Foxo3a protein mutated at S^644^ translocated into the nucleus and activated the transcription of the Fas-L and p21^Cip^ genes. This, in turn, inhibited leukemic cell proliferation and induced apoptosis. These and the preceding observations may be interpreted to mean that in AML, IKK signaling takes control and replaces former signaling events. Whether this takeover of PI3K signaling by IKK is a general feature or is restricted to AML cells is unknown. The latter option would suggest a direct role of DNA damage in the origin of AML.

## 14. Sequential Activation of the AKT/IKK/NF-κB Pathway and Its Role in Cancer Metastasis and Angiogenesis

NF-κB activation via sequential activation of PI3K, AKT, and IKK_β_ is evident in a report by Agarwal et al. [70]. They studied two (SW480 and RKO) out of five CRC cell lines that displayed constitutive activation of AKT and both NF-κB- and β-catenin-dependent transcription. Inhibition of the transcription of NF-κB targets was demonstrated by WT PTEN, KD AKT, and KD IKK_β_, as well as by siRNA targeting of AKT and IKK_α_. Either WT PTEN, KD AKT, or KD IKK_α_ (a factor of the alternate pathway) inhibited both NF-κappaB and β-catenin transcription, whereas KD IKK_β_, although it inhibited NF-κappaB, did not have any effect on β-catenin expression in RKO and SW480. It is likely that Foxo3a deactivation would respond in the same way to the silencing of each of these members of the PI3K pathway. However, Foxos were unfortunately not examined in this report.

An array-based gene expression analysis of cell lines with constitutive expression of NF-κB and β-catenin following inhibition by KD IKK_α_ or WT PETEN showed reductions in the expression of multiple genes implicated in angiogenesis and metastasis. Some of these genes are dependent on NF-κB or β-catenin or both. IL-8, μPA, and VEGF-C are dependent on both. Elevated expression of mRNA for these genes is observed in a substantial fraction of colorectal tumors compared to matched normal mucosa samples (a two-fold increase in IL-8 expression in about 85% of colon and 75% of rectal tumors, whereas μPA is elevated in 75% of colon and 79% of rectal tumors).

Other reports suggest a more complex interplay between the intermediate targets of the pathway, as some cooperation between intermediate targets rather than a strict linear sequence of activation could be at play. Nevertheless, activation culminates in the final activation of NF-κB via IKK [71].

Thus, returning to the dilemma raised above regarding AML, the independent signaling of IKK in the previous steps of the PI3K pathway suggests that ATM-NEMO-IKK signaling is exclusively responsible for NF-κB activation in AML, which emphasizes the primary role of DNA damage in AML. A similar reliance of Foxo regulation on IKK independently from other members of the PI3K pathway was also observed in AML by Chapuis et al. [69], who raised a question regarding the extent of the coupled and simultaneous regulation of Foxo and NF-κB in tumorigenesis. Synergy between Foxos and NF-κB may blur the attribution of effects to either one specifically.

Tumor invasion through NF-κB activation has been associated with both canonical and non-canonical pathways. In addition, it can be mediated through sequential activation of the pathway or the activation of a single target at the converging downstream end, such as any of the IKK proteins. This route appears to be available to scaffold proteins, a class of proteins that regulate a signaling node in order to determine the choice of a downstream target. This has been shown for the scaffold protein connector enhancer of KSR (CNK), which facilitates Raf activation in the Ras-Raf-MAPK/ERK kinase (MEK)-ERK pathway. CNK1 can regulate the ERK and JNK pathways; however, it was shown that CNK1 mediates human breast and cervical cancer via cooperation with the non-canonical NF-κB pathway, independently of the ERK and JNK pathways [72].

To complicate matters further, positive feedback between FOXC1 and NF-κB has been described in the basal type of breast cancer (BLBC). EGFR upregulates FOXC1 expression in BLBC and prostate cancer cells through the Ras/ERK and PI3K pathways. Overexpression of the p65 subunit of NF-κB in the MDA-MB-468 cell line (but not in the MDA-MB-231 cell line, which has a much lower level of EGFR expression) markedly increased FOXC1 promoter activity. A positive feedback regulatory loop between FOXC1 and NF-κB has been demonstrated in BLBC, which may explain why both proteins are highly specific to BLBC. FOXC1 requires NF-κB to regulate cell migration and invasion [73,74], as seen in many other tumor types. It has also been reported that FOXC1 controls the stemness of basal-like breast cancer via Hh signaling, but further work is required to elucidate the signaling sequence [75].

## 15. A Collateral Note on the ATM-NEMO-NF-κB Pathway

A final question raised by these observations concerns an unsuspected parallelism in the oncogenic mechanisms of AML and BRCA-induced breast cancer. Frequent DNA damage is thought to be caused by homology-directed repair or homologous recombination (HR), a mechanism linked to the S phase of the cell cycle, which is prevalent in highly proliferating cells, such as hematopoietic progenitors and regenerating mammary cells. The BRCA tumor suppressor associated with breast cancer participates in homology-directed DNA repair and is involved in DNA repair in epithelial cells of the regenerating mammary gland. Thus, the underlying mechanism of breast cancer promotion by BRCA appears to be the same or closely related to that of AML cells. Enhanced cell proliferation-associated DNA damage triggers the ATM-NEMO-IKK pathway, and NF-κB could be the final target for tumorigenesis in both diseases.

## 16. Multifaceted Involvement of NF-κB in Tumorigenesis

NF-κB seems to be involved in all stages of tumorigenesis, ranging from initiation thanks to its role in chronic inflammation, to the induction of the invasive phenotype, EMT, and stemness [76,77]. Curiously, no oncogenic mutations of IKKs have been found. In the regulation of EMT and stemness, NF-κB seems to act at a common converging node of signaling pathways such as PI3K/AKT, as suggested by its cooperation with FOXOS or the ability of a super-repressor form of I_κ_B_α_ or genetic silencing of RelA to suppress Ras-mediated tumorigenesis [78]. It is difficult to envisage how such a vast array of functions could be mediated by a factor or restricted group of factors. Interestingly, a protein that interacts with some of the downstream effectors of these pathways, Rap1, has also been implicated in invasiveness and especially in stemness, which could be fundamental to immortalization.

## 17. Possible Downstream Effectors of Telomerase Reactivation in Transformation

In a former paper [9], we hypothesized that Rap1, a protein that associates indirectly with telomeres via interaction with the sheltering complex protein TRF2, might be involved in the coupling of telomere damage with oncogenic signaling. More recently, we became aware of other publications that strongly implicate Rap1 in transformation. The E6 protein of high-risk papilloma viruses HPV16 and HPV18 binds several proteins in addition to p53. One of them is E6TP1 (E6-targeted protein), a Rap GTPase-activating protein that negatively regulates mitogenic signaling mediated by Rap. The ability of E6 mutants to bind and degrade E6TP1 (Figure 3) correlates better with the E6-induced immortalization of mammary epithelial cells (MECs) than the ability to degrade p53. The correlation of E6TP1 degradation with the immortalization of MECs exceeds that of mutated p53. Among a panel of E6 mutants, all of those capable of immortalizing MECs were able to induce telomerase activity. One mutant, previously reported to not activate telomerase in a transient expression system, was shown to induce telomerase activity after an analysis of immortalized MECs [79]. E6TP1 shows high sequence homology with Rap1 GAP, which negatively regulates mitogenic signaling by Rap1. Presumably, E6 can also degrade Rap GAP (Figure 3), and it is therefore likely that other molecules that degrade RapGAP could also stimulate Rap mitogenic activity and recapitulate E6-induced telomerase activity and MEC immortalization.

## 18. Interplay Between Telomere Shortening and Rap1 (Figure 3)

Rap1 associates with telomeres through TRF2, as it does not directly bind DNA. Rap1’s functions at telomeres have been challenging to elucidate. Its best-characterized function is the regulation of homology-directed repair, a pathway that facilitates telomere sister chromatide exchange unless inhibited by Rap1.

Rap1 loss may increase telomere shortening; conversely, a decrease in telomere length increases Rap1 shuttling and binding to non-telomeric genomic sites. This may facilitate either Rap1 interaction with other DNA-binding proteins, such as NF-κB and FOXOs, or Rap1 shuttling to the nuclear or cytoplasmic matrix, where this interaction can take place [80]. Blanco et al. found that TRF2 overexpression in the absence of telomerase activity leads to critically short telomeres and increased epithelial carcinogenesis, suggesting that carcinogenesis could be related to the disruption of TRF2-Rap1 interaction with the release of non-telomeric Rap1 [81].

As Rap1 has been shown to form a complex with IKKs (Figure 3) and phosphorylate p65, thereby rendering NF-κB transcriptionally competent, it is possible that Rap1 is the final effector of many of the oncogenic signaling pathways described above. Furthermore, there are NF-κB binding sites in the Rap1 promoter. This and other findings suggest the existence of a feedback loop between these factors [82,83,84].

## 19. Interaction of Rap1 and Zscan4 (Figure 3)

Lee et al. described an interaction of Rap1 with the newly identified embryonic stem cell marker Zinc-finger and SCAN domain containing 4 gene (Zscan4) (Figure 3), which performs a key function in genomic stability by regulating telomere elongation. These authors analyzed two cell lines, MCF7 and SaOS2, epithelial/telomerase+ and mesenchymal/telomerase−, respectively, and found that Zscan4 was present in cancer cells from both cell lines. Rap1 bound to the C-terminal domain of Zscan [85]. Transfection of cancer cells with Rap1 siRNA resulted in significantly reduced expression of Zscan4, whereas overexpression of Rap1 gave rise to greater Zscan4 expression [85]. It is plausible that Zscan4 could replace reprogramming factors that convert committed cells into pluripotent stem cells, triggering the final step of transformation. In this way, neoplastic transformation would require a surplus of non-telomeric Rap1 facilitated by the repression of Rap1 GTPase (RapGAP) or telomere shortening/damage.

The interleukin-1 receptor accessory protein (IL1RAP) is a coreceptor of interleukin-1 receptor type1. IL1RAP expression is higher on the GMP population compared with other progenitor populations and HSCs. It is not expressed on normal human HSCs but is highly expressed on primitive CD34^+^ CD38^−^ CML cells. Again, this fact suggests that leukemic blast cells are derived from progenitors rather than directly from HSCs and that IL1 RAP upregulation might be related to increased non-telomeric RAP [86].

Some reports support the role of RAP1 as a converging node of several oncogenic pathways.

In a study of neural gliomas, Niola et al. [87] showed that Id proteins repress Rap1GAP with concurrent activation of Rap (Figure 3). Activation of Rap1 through the Id-mediated repression of Rap GAP leads to the emergence of stemness features, such as the stem cell marker Nestin and neurosphere generation, as well as adhesion to the endothelial niche. Conversely, Id depletion facilitates immediate release from the niche and the expression of differentiation markers. Multipotent NSCs in the ventricular zone of the brain are anchored to the ventricular surface by an apical membrane. Neural progenitors acquire RapGAP expression as soon as they lose contact with the ventricular surface. Id deletion is associated with Rap1GAP expression and disruption of adhesion to the niche. Interestingly, these authors showed that the ID-RAP1 axis is especially relevant for the mesenchymal type of high-grade glioma [88].

Rap1 GAP has been found to be lost or downregulated in various types of tumors and at a high frequency in thyroid tumors. Rap1 GAP deficiency correlates with increased cell migration and invasiveness [89]. In oropharyngeal squamous carcinoma, active GTP-bound Rap1 was upregulated compared to normal or immortalized keratinocytes. Inactivation of Rap1 by Rap1GAP resulted in strong tumor inhibition [90]. Rap1GAP was also identified as a tumor suppressor in pancreatic cancer [91]. The effects of tetratricopeptide repeat domain 17 (TTC17) downregulation on the invasiveness and metastasis of breast cancer were shown to be linked to activation of the Rap1/CDC42 pathway [92].

## 20. Further Comments

In this study, we focus on oncogenic signaling cascades that initiate in normal progenitor cells. The regenerative ability of early progenitors is maintained by stem cell-associated telomerase. Nevertheless, sustained proliferation is usually followed by exhaustion of regenerative potential. This is at odds with the long-lasting, persistent proliferative ability observed in fully differentiated cells induced to de-differentiate by the four factors (iPSCs) and in cancer cells. Thus, we hypothesize that the development of cancer involves the de-differentiation of a post-stem cell that resorts to a different type of telomerase reactivation or a mechanism of telomere maintenance that differs from the one that characterizes normal stem cells.

The GSK 3 node appears to be crucial in the regulation/deregulation of growth in tissues endowed with a high proliferation ability, such as intestinal tissue or blood. Within chromosomal translocation-associated leukemia, those involving MLL fusion proteins characteristically affect cells in a very early differentiation stage showing, immature features and high proliferating activity. MLL fusion proteins activate a self-renewal program apparently initiated by the HoxA9 and Meis1 genes. These genes are, under physiological conditions, only expressed in c-Kit+ and Thy^lo^ Lin^lo/−^ Sca1+ rhodamine 123^lo^ HSCs, and their expression is rapidly downregulated in more differentiated multipotential common lymphoid and myeloid progenitors. However, in MLL leukemias, there is persistent expression of Hoxa7, Hoxa9, and Meis1 downstream of the stem cell stage. Interestingly, Hoxa9 and Meis are upregulated in advance of the full self-renewal signature. In vitro growth of MLL-AF9-transduced human cord blood cells was shown to be strictly dependent on the Flt3 ligand. This indicates that the cancer stem cell and its normal precursor expressed FLT3, which demonstrates that they are located downstream of the HSCs [9]. Several facts indicate the involvement of the GSK3 node in oncogenesis in these and other immature leukemias. For instance, a reversible myelomonocytic block in MLL-ENL leukemia was shown to be dependent on c-myc. A dominant negative myc mutant prevented the block caused by MLL-ENL and precluded transformation [9]. MLL-transformed cells were shown to be highly dependent on GSK3 by culturing MLL-transduced myeloid progenitors with a GSK3 inhibitor that reduced their clonogenic potential and proliferation in contrast to cells immortalized by other fusion proteins that did not show adverse effects [93]. However, translocation products from AML-ETO, PML-RARα, and PLZF-RARα can activate Wnt signaling via the induction of plakoglobin (γ-catenin), which blocks β-catenin degradation and activates TCF, LEF, and their target c-Myc, thus enhancing cell proliferation and the preservation of immature features [9].

## 21. Impacts of Distinct Oncogenes on Cells at Different Developmental Stages

Tumor differentiation markers can be used as an indication of the origin of cancer from a specific developmental stage of the corresponding cell lineage. In the mammary gland, keratin6 and Sca-1 are preferentially expressed by mammary stem or progenitor cells, even if they are not restricted to these compartments. As in the intestine and other tissues, the Wnt pathway physiologically activates cells in the early developmental stages. Experiments on transgenic mice showed [94] that overexpression of Wnt-1 or its downstream effectors β-catenin or c-Myc under the control of the MMTV promoter resulted in development of hyperplastic mammary glands that mainly comprised progenitor cells and mammary tumors derived from these cells. The early markers Keratin 6 and Sca-1 were detected in many hyperplastic ducts and mammary tumors of Wnt-1 transgenic mice but were not increased in premalignant ducts or tumors in MMTV-Neu, MMTV-Hras, or MMTV-PyMT transgenic mice. IH staining demonstrated that Wnt-1-induced tumors were composed of luminal and myoepithelial cells (both cell types appear to be malignant in Wnt-1-induced tumors), whereas the MMTV-β-catenin and MMTV-c-Myc tumors showed a hyperplastic myoepithelial component and malignant luminal cells. In contrast, myoepithelial hyperplasia was absent in MMTV-Neu-, MMTV-Hras-, and MMTV.PyMT-induced tumors. The coexistence of myoepithelial and luminal cells in Wnt-1-induced tumors suggest transformation from a common progenitor. Some of them arose in Pten-heterozygous MMTV-Wnt-1 transgenic mice, and the wild-type Pten locus was missing in both luminal and myoepithelial cells. Implication of the Pten pathway in tumors induced by the Wnt pathway may be facilitated by the above-mentioned crosstalk between these pathways and the role of the GSK3β node. The hyperplastic myoepithelial component, which is not yet transformed in MMTV-β-catenin and MMTV-c-Myc, may correspond to the adenomatous phase observed in intestinal adenomas that ultimately gives rise to adenocarcinomas. The authors’ interpretation is that deregulated Wnt signaling causes excessive proliferation and arrest early mammary development, consistent with our own interpretation of a partial block in intestinal Wnt-deregulated signaling. Other studies [95] have shown that Neu oncogene-induced tumors have a uniform luminal histology, require CDK4 and cyclin D1 for transformation, and reflect the Erb2 subtype of human cancer. In contrast, Wnt-1-driven tumors exhibit features in common with the human basal subtype that falls within the group of (TN) tumors (lacking ER and PR expression and Her2 gene amplification). Id1 and Id3 expression, which reflect an immature stage, is observed in the tumor cells of Wnt-1-driven tumors but not in Neu-induced tumor cells.

The phenotypic difference in tumors arising in MMTV-Wnt1 and MMTV-β-catenin and MMTV-c-myc models suggests a cell of origin in progenitors that, in the case of MMTV-Wnt1, belongs to an earlier developmental stage. In these tumors, the more immature component is already malignant, whereas in the other two tumor types, it is adenomatous. Interestingly, immaturity is associated with the appearance of PTEN loss of heterozygosity, which was not detected in the other transgenics. Pathway deregulation in Wnt1 transgenics occurs earlier than in the other two, at a step that may involve the GSK3-β node and a wider range of downstream targets, including Notch activation, which is responsible for a more undifferentiated phenotype.

Lim et al. provided a broader stratification of the developmental stages of cancer origination [96]. These authors prepared cell suspensions from reduction mammoplasties from normal females or BRCA mutation carriers. After discarding hematopoietic and endothelial cells, they fractionated the resulting Lin^−^ population into four distinct subsets with the help of two antibodies against CD49f (α6 integrin) and EpCAM (also referred to as CD326 (against epithelial cell adhesion molecule)). EpCAM was predominantly expressed on luminal cells, whereas CD49f was expressed on basal cells. More specifically, the CD49f^hi^ EpCAM^−^ subpopulation expressed the basal lineage markers p63, cytokeratin 14, and vimentin but did not express the estrogen receptor or progesterone receptor. In contrast, the CD49f^−^ EpCAM^+^ and CD49f^+^ and CD49f^+^ EpCAM^+^ subpopulations expressed luminal markers including cytokeratin 8/18, cytokeratin 19, GATA-3 (a regulator of luminal differentiation), and Mucin-1 (MUC-1). The CD49f^−^ EpCAM^+^ fraction should contain mature luminal cells because there are far fewer estrogen- and progesterone receptor-negative cells in this fraction. Only the CD49f^+^ EpCAM^+^ subset contained a high proportion of cells positive for CD133 (prominin-1), a progenitor marker in diverse tissues. The CD49f^−^ EpCAM^−^ fraction mainly comprised stromal fibroblasts. Only the CD49f^hi^ EpCAM^−^ subset (also referred to as the MaSC-enriched population) showed a capacity for mammary regeneration. This latter population was substantially reduced in BRCA carriers, whereas the luminal progenitor subpopulation (CD49f^+^ EpCAM^+^) was increased in them relative to normal breast tissue. Gene expression signatures derived from microarray studies disclose the association of the luminal progenitor signature with basal cancers and that of the mature luminal signature with the luminal A and B subtypes. The MaSC signature was more concordant with the normal-like and newly, tentatively defined claudin-low subtypes. We postulate that these latter subtypes arise from progenitors at an earlier stage than basal cells. Their cell of origin is difficult to establish, as it might be reflective of a heterogeneous population including basal cells and myoepithelial cells. Furthermore, they do not have a clear counterpart in clinical classifications, although they could be grouped with poor outcome groups such as LumB, basal-like, and HER2+/ER−, all of which might arise in early developmental stages.

An interesting finding that aligns with the findings noted above concerns the correlation of oncogenic pathways with distinct stages of presumed cell origin: PTEN LOH is an event correlating with basal-type tumors, and TP53 loss predominates in luminal tumors [97].

Another interesting aspect of secondary oncogenic pathways is the advent of some specific mutations that apparently endow tumor cells with further initiating capacity and are detected when tumors reach the clinical stage defined by metastasis. In breast cancer, Id1 was identified as the only transcription factor within a set of genes associated with high risk of lung metastasis. A monospecific monoclonal antibody revealed positive staining for Id1 in 36% of TN (triple-negative) breast cancers. This proportion is higher in the metaplastic subtype. Among non-TN breast tumors, only 1 out of 105 was positive for Id1 expression. This secondary mutation may be dependent on the primary oncogenic pathway, as Id1 expression was detected in 45% of lung metastases in Er- Pr- cases and 0 out of 16 hormone receptor-positive cases. Knockdown of Id1 and Id3, either individually or in combination, from a cell line representative of the TN human subgroup of breast cancer resulted in partial or complete inhibition of the tumor-initiating capacity, respectively. Id1/Id3 knockdown suppressed the lung metastatic potential of the selected subline. In a Wnt-driven tumor, knockdown of Id1 and Id3 resulted in significant impairment of in vitro mammosphere generation and tumor initiation. The same knockdown did not cause alterations in Neu-induced tumors; mammosphere formation, which is a measure of tumor-initiating capacity, is lower in Neu tumors but is not decreased by the knockdown of Id1 and Id3 [95].

As oncogenes usually impact differentiation during tissue turnover, a correlation between oncogenic signaling pathways and the lineage developmental stage is expected. Evidently, the oncogenic signaling pathways reviewed here comprise but a fraction of those used by tumors, which, in addition to their crosstalk, renders cancer research highly complex and challenging. Fortunately, it is likely that most of these pathways converge on a few common targets that are essential for driving immortalization. For instance, Ha-Ras or c-Neu can transactivate the peptidyl-prolyl isomerase Pin1 through E2F [98]. In turn, Pin1 can stimulate NF-κB activity. It accomplishes this effect by inhibiting the binding of p65 to I_κ_B, which results in the nuclear accumulation of p65/p50 [99].

## 22. Conclusions

In former publications, we have advocated the idea that cancers originate predominantly in cells engaged in tissue turnover rather than directly from normal stem cells. Stem cell proliferation exhaustion and conditions of tissue atrophy [7] that plausibly cause telomere shortening usually precede cancer development. On the other hand, cancers that appear to be directly derived from stem cells like those arising in the Beckwith–Wideman Syndrome tend to manifest concurrently in different organs and exhibit inherent alterations in telomere complex function [9]. These observations in conjunction with the sudden change in telomerase expression observed in an adenoma-carcinoma sequence where telomerase apparently recovers the ability of balancing telomere loss and cell proliferation, a feature of cancer stem cells, suggest that a previous process of telomere erosion/damage has paved the way for telomerase re-expression. Distinct signaling pathways initiated at different stages of cell differentiation may contribute to further telomere erosion/damage while converging on common downstream targets such as IKK and NFkB, which in cooperation with Rap1, released from shortened/damaged telomeres, induce expression of the embryonic stem cell marker Zscan4 and immortalization. In this process, telomere maintenance will rely, most likely, on embryonic mechanism/s of self-renewal bypassing the normal stem cell mechanism of telomerase control.

## Data Availability

No new data were created or analyzed in this study. Data sharing is not applicable to this article.
